# Phytochemicals and Biological Activity of Desert Date (*Balanites aegyptiaca* (L.) Delile)

**DOI:** 10.3390/plants10010032

**Published:** 2020-12-25

**Authors:** Hosakatte Niranjana Murthy, Guggalada Govardhana Yadav, Yaser Hassan Dewir, Abdullah Ibrahim

**Affiliations:** 1 Department of Botany, Karnatak University, Dharwad 580003, India; hnmurthy60@gmail.com (H.N.M.); govardhanyadavgs@gmail.com (G.G.Y.); 2 Plant Production Department, P.O. Box 2460, College of Food and Agriculture Sciences, King Saud University, Riyadh 11451, Saudi Arabia; adrahim@ksu.edu.sa; 3 Faculty of Agriculture, Kafrelsheikh University, Kafr El-Sheikh 33516, Egypt

**Keywords:** bioactive compounds, polysterols, polyphenols, saponins, therapeutic properties

## Abstract

Many underutilized tree species are good sources of food, fodder and possible therapeutic agents. *Balanites aegyptiaca* (L.) Delile belongs to the Zygophyllaceae family and is popularly known as “desert date”, reflecting its edible fruits. This tree grows naturally in Africa, the Middle East and the Indian subcontinent. Local inhabitants use fruits, leaves, roots, stem and root bark of the species for the treatment of various ailments. Several research studies demonstrate that extracts and phytochemicals isolated from desert date display antioxidant, anticancer, antidiabetic, anti-inflammatory, antimicrobial, hepatoprotective and molluscicidal activities. Mesocarp of fruits, seeds, leaves, stem and root bark are rich sources of saponins. These tissues are also rich in phenolic acids, flavonoids, coumarins, alkaloids and polysterols. Some constituents show antioxidant, anticancer and antidiabetic properties. The objective of this review is to summarize studies on diverse bioactive compounds and the beneficial properties of *B. aegyptiaca*.

## 1. Introduction

**Figure 1:**
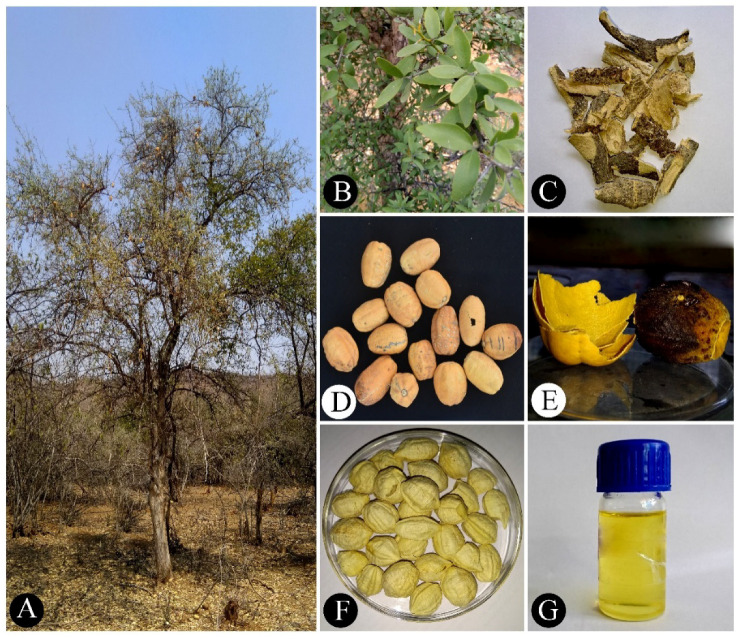
Morphology of *Balanites aegyptiaca* (L.) Delile: (**A**) habit, (**B**) leaves, (**C**) stem bark, (**D**) ripened fruits, (**E**) rind (left) and pulp (right), (**F**) seed kernels and (**G**) seed oil.

*Balanites aegyptiaca* (L.) Delile (Family: Zygophyllaceae) is an underutilized fruit-yielding tree (Figure 1A) native to Africa and distributed in tropical and subtropical regions of Africa, from Senegal in the west (16 °W) to Somali in the East (49 °E) and Jordan in the north (35 °N) to Zimbabwe in the south (19 °S). *B. aegyptiaca* is also distributed in India, Myanmar, Iran, Jordan, Oman, Palestine, Saudi Arabia, Syria and Yemen [1]. Young leaves (Figure 1B) and tender shoots are used as vegetables. Leaves and fruits are used as fodder for livestock [1]. Fiber obtained from tender bark and older dried bark is used for the preparation of medicines (Figure 1C). Unripe and ripe fruits (Figure 1D,E) are edible and popularly known as “desert date”. The fruits are processed into beverages and liquor. Timber is suitable for the construction of furniture, domestic items and musical instruments. The wood produces high-quality charcoal fuel and industrial activated charcoal. Gum or resin produced from stems are used as glue. Seeds contain about 49% edible oil (Figure 1F,G), which is also used in the production of biodiesel fuel [1].

*B. aegyptiaca* is used in African and Indian traditional medicine. Roots and bark are purgative and anthelmintic. A decoction of roots is used to treat malaria. The bark is used to deworm cattle, and the roots are boiled into a soup and used to treat edema and stomach pains. Roots are also used as an emetic [1]. The fruit is used to treat jaundice in Sudan [2]. Seed oil is used as a laxative and for the treatment of hemorrhoids, stomach aches, jaundice, yellow fever, syphilis and epilepsy [3]. Bark extracts are used to kill freshwater snails and copepods. A decoction of bark is also used as an abortifacient and antidote in West African traditional medicine [4]. 

## 2. Nutritional Composition of Fruits, Seeds and Leaves

Ripe fruits display a thin brownish epicarp (Figure 1E), dark brown and fleshy mesocarp (Figure 1E) and thick endocarp nut (Figure 1F). The edible parts of the pulp and kernel yield oil. The pulp is rich in carbohydrates (62.63%) and protein (9.19%; Table 1) [5]. Fruit pulp shows lesser amounts of fat (2.58%) and dietary fiber (2.93%). The overall energy value is 346.82 kcal/100 g. Fruits are also rich in minerals, including calcium, magnesium, phosphorus, potassium and sodium (Table 1) [6]. Iron, copper, manganese, lead, chromium, cobalt, cadmium and selenium are reported in lower concentrations (Table 1). Major fatty acids in fruit pulp are oleic (37.17%), linoleic (27.73%) and palmitic (22.02%; Table 2) [7]. The fruit pulp also exhibits amino acids (Table 3) [8] and vitamins (Table 3). Antinutritional factors are comparatively less (Table 4) [5].

Seeds are rich in fixed oil content (49.00%) with a significant content of proteins (32.40%) and carbohydrates (8.70%; Table 1) [9,10]. Seed oil is used for edible purposes; major fatty acids are linoleic (47.84%), oleic (22.80%), palmitic (16.68%) and stearic (11.67%) (Table 2) [11]. It has been demonstrated that biodiesel from seed oil meets all international biodiesel standards [11]. Seeds contain minerals, such as potassium, phosphorus and calcium in higher concentrations (Table 1) and amino acids (Table 3) [9]; seed cake is used for animal feed. However, seeds also contain oxalate (8.51 mg/g DW), antinutrient and possibly toxic constituents (Table 4).

**Table 1:** Nutritional and mineral composition of desert date pulp, seeds and leaves.

Proximate (%)			
Components	Pulp [5,6]	Seeds [9,10]	Leaves [12,13]
Moisture	18.27	5.20	13.11
Protein	9.19	32.40	15.86
Fat	2.58	49.00	2.90
Ash	4.40	3.30	9.26
Carbohydrate	62.63	8.70	28.12
Dietary fiber	2.93	1.40	30.75
Energy (kcal/100g)	346.82	605.40	202.02
Mineral composition (mg/g DW)			
Calcium (Ca)	1.41	1.51	0–2.65
Iron (Fe)	0.0494	0.0484	NR *
Magnesium (Mg)	0.73	0.887	0.23–0.77
Phosphorus (P)	0.48	3.60	1.51–5.32
Potassium (K)	22.20	6.36	1.76–4.81
Sodium (Na)	0.48	0.053	NR
Zinc (Zn)	0.0065	0.0286	NR
Copper (Cu)	0.0039	0.0118	0.05–0.65
Manganese (Mn)	0.0033	0.0192	0.02–0.08
Lead (Pb)	0.0030	0.0050	NR
Chromium (Cr)	0.0040	0.0060	NR
Cobalt (Co)	0.0107	0.0120	NR
Cadmium (Cd)	0.0347	0.0163	NR
Selenium (Se)	0.0005	NR	NR

* NR = not reported.

Young leaves and shoots are used as vegetables in African countries. Leaves and shoots are also popular livestock fodder [1]. Leaves are a good source of carbohydrates (28.12%) and proteins (15.86%) and contain ash (9.26%) and dietary fiber (30.75%; Table 1) [12]. Leaves also provide minerals (Table 1), fatty acids (Table 2), amino acids (Table 3) and vitamins (Table 3) [12,13]. Leaves contain antinutrients in meager concentrations (Table 4).

**Table 2:** Fatty acid composition of desert date pulp, seeds and leaves.

Fatty Acid	Pulp (% in oil) [7]	Seeds (% in oil) [11]	Leaves (µg/g DW) [13]
Saturated fatty acids (SFA)			
Lauric (C12:0)	ND *	ND	0.17–0.28
Myristic (C14:0)	0.1	0.05	0.043–0.074
Pentadecylic (C15:0)	ND	0.046	ND
Palmitic (C16:0)	22.02	16.683	0.29–1.98
Margaric (17:0)	ND	0.106	ND
Stearic (C18:0)	ND	11.67	0.008–0.049
Nonadecylic(19:0)	ND	0.032	ND
Arachidic (C20:0)	ND	0.34	0.274–0.439
Behenic (C22:0)	ND	0.059	0.026–0.038
Tricosylic (C23:0)	ND	0.012	0–0.003
Lignoceric (C24:0)	ND	0.042	0.028–0.298
Hyenic (C25:0)	ND	ND	0.078–0.121
Monounsaturated fatty acids (MUSFA)			
Pentadecenoic (C15:1)	ND	0.003	0.08–0.31
Palmitoleic (C16:1)	ND	0.027	ND
Oleic (18:1)	37.17	22.807	0.03–0.061
Nonadecenoic (19:1)	ND	0.175	ND
Gadoleic (20:1)	ND	0.061	ND
Tetracosenoic (C24:1)	ND	ND	0.077–0.266
Polyunsaturated fatty acids (PUSFA)			
Hexadecadienoic (C16:2)	ND	ND	0.406–1.835
Linoleic (C18:2)	27.73	47.847	0.025–0.642
Eicosadienoic (C20:2)	ND	ND	0.116–0.296
Hexadecatrienoic (C16:3)	ND	ND	0.761–2.142
Octadecatrienoic (C18:3)	ND	ND	0.20–0.525
Total SFA (%)	22.16	29.04	1.84–3.38
Total MUSFA (%)	37.17	23.073	0.179–0.637
Total PUSFA (%)	27.73	47.847	1.727–5.174

* ND = not detected.

**Table 3:** Amino acid and vitamin composition of desert date pulp, seeds and leaves.

Amino Acid	Pulp (mg/g DW) [5,8,14]	Seeds (g/100 g of Protein) [9]	Leaves (g/100 g of Protein) [12,13]
Alanine	2.90	3.50	1.80
Aspartic acid	4.43	8.29	7.86
Arginine	3.00	2.70	4.20
Cystine	1.65	2.52	0.79
Glutamic acid	7.10	8.91	10.80
Glycine	2.52	4.10	9.65
Histidine	0.80	1.99	2.83
Isoleucine	1.87	3.47	3.50
Leucine	3.04	6.47	6.23
Lysine	1.64	5.00	4.51
Methionine	0.60	0.75	0.73
Phenylalanine	1.90	4.61	4.80
Proline	30.80	2.78	1.85
Serine	1.80	4.29	2.01
Threonine	2.17	4.25	2.88
Tyrosine	1.84	2.75	3.16
Valine	2.23	3.29	4.07
Tryptophan	0.70	NR *	NR
Vitamin (mg/g DW)			
Vitamin A			
α-carotene	NR	NR	0.33–0.54
β-carotene	0.6484	NR	0.25–0.81
β-cryptoxanthin	NR	NR	0.02–1.14
Vitamin B			
Thiamine	0.0027	NR	0.24–0.51
Riboflavin	0.0007	NR	NR
Niacin (B3)	0.0174	NR	NR
Vitamin B6	0.0021	NR	NR
Vitamin C			
Ascorbic acid	1.05	NR	0.57–2.05
Vitamin E			
α-tocopherol	NR	NR	0.08–0.57
β-tocopherol	NR	NR	0.01–0.04
γ-tocopherol	NR	NR	0.01–0.063
δ-tocopherol	NR	NR	0.13–0.96
Vitamin K			
Phylloquinone	NR	NR	0.21–1.37

* NR = not reported.

**Table 4:** Antinutritional components of desert date pulp, seeds and leaves.

Antinutritional Factors (mg/g DW)			
Components	Pulp [5]	Seeds [9]	Leaves [12]
Oxalate	0.38	8.51	0.75
Phytate	1.82	0.2133	0.0297
Tannins	0.40	0.275	0.041
Saponin	0.62	4.279	ND
Nitrate	ND *	0.39	ND
Cyanogenic glycosides	ND	0.0096	ND

* ND = not detected.

## 3. Phytochemicals Isolated from Desert Date

*B. aegyptiaca* produces a variety of secondary metabolites, such as polyphenols (phenolic acids, flavonoids and coumarins), alkaloids, steroids, saponins (spirostanol saponins, furostanol saponins and open-chain steroidal saponins) and pregnane glycosides, isolated from plant tissues, such as fruit, seeds, leaves, stem bark, roots and galls (Table 5).

### 3.1. Polyphenols

Polyphenols exhibit phenolic structural features with one or more aromatic rings, each with one or more hydroxyl groups [15]. Polyphenols are grouped into phenolic acids, flavonoids, stilbenes, lignans and tannins. These compounds are important as natural therapeutic agents involved in the prevention of degenerative diseases, particularly cancers, cardiovascular diseases and neurodegenerative diseases [16]. 

**Figure 2:**
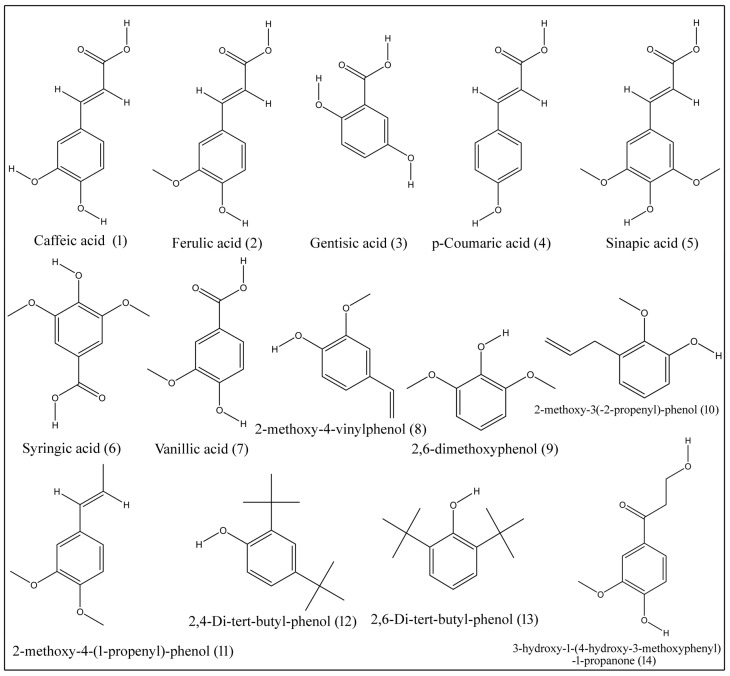
Structures of phenolic compounds isolated from desert date.

Phenolic acids are nonflavonoid polyphenolic compounds of benzoic acid and cinnamic acid. Major phenolic acids, which are isolated from tissues of *B. aegyptiaca*, include caffeic acid (**1**), ferulic acid (**2**), gentisic acid (**3**), p-coumaric acid (**4**), sinapic acid (**5**), syringic acid (**6**), vanillic acid (**7**), 2-methoxy-4-vinylphenol (**8**), 2,6-dimethoxyphenol (**9**), 2-methoxy-3(-2-propenyl)-phenol (**10**), 2-methoxy-4-(1-propenyl)-phenol (**11**), 2,4-di-tert-butyl-phenol (**12**), 2,6-di-tert-butyl-phenol (**13**) and 3-hydroxy-1-(4-hydroxy-3-methoxyphenyl)-1-propanone (**14**) (Table 5; Figure 2) [2,17,18,19]. 

### 3.2. Flavonoids

**Figure 3:**
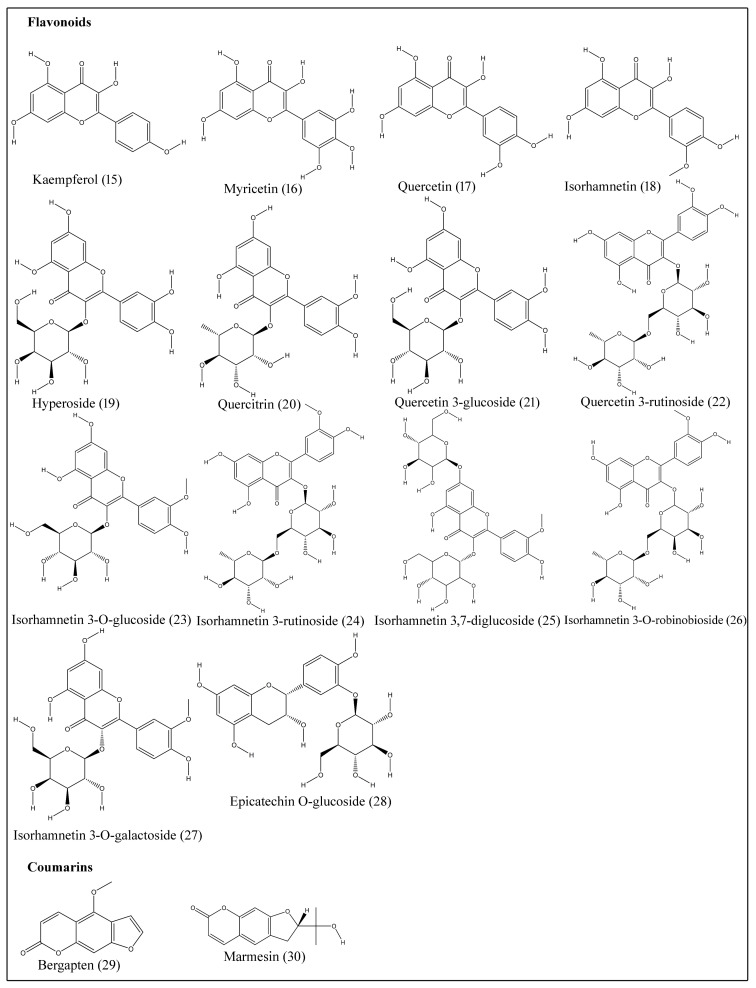
Structures of flavonoids and coumarins isolated from desert date.

Flavonoids exhibit a diphenyl propane–flavone skeleton with a three-carbon bridge between phenyl groups and commonly cyclized with oxygen. Epicatechin O-glucoside (**28**), hyperoside (**19**), isorhamnetin (**18**), isorhamnetin-3-O-glucoside (**23**), isorhamnetin 3,7-diglucoside (**25**), isorhamnetin 3-O-galactoside (**27**), isorhamnetin 3-O-robinobioside (**26**), isorhamnetin 3-rutinoside (**24**), kaempferol (**15**), myricetin (**16**), quercetin (**17**), quercetin 3-glucoside (**21**), quercetin 3-rutinoside (**22**) and quercitrin (**20**) are isolated from different tissues of *B. aegyptiaca* (Table 5; Figure 3) [19,20,21,22].

**Table 5:** Phytochemicals isolated from various parts of desert date.

No.	Compound Name	Plant Parts	Reference
**PHENOLICS**			
1	Caffeic acid	Gall, leaf	[19]
2	Ferulic acid	Gall, leaf	[19]
3	Gentisic acid	Gall, leaf	[19]
4	p-Coumaric acid	Gall, leaf	[19]
5	Sinapic acid	Gall, leaf	[19]
6	Syringic acid	Stem bark	[2]
7	Vanillic acid	Stem bark	[2]
8	2-methoxy-4-vinylphenol	Fruit, leaf	[18]
9	2,6-dimethoxyphenol	Leaf	[18]
10	2-methoxy-3(-2-propenyl)-phenol	Leaf	[18]
11	2-methoxy-4-(1-propenyl)-phenol (Isoeugenol)	Leaf	[18]
12	2,4-di-tert-butyl-phenol	Seed	[17]
13	2,6-di-tert-butyl-phenol	Seed	[17]
14	3-hydroxy-1-(4-hydroxy-3-methoxyphenyl)-1-propanone	Stem bark	[2]
**FLAVONOIDS**			
15	Kaempferol	Leaf	[19]
16	Myricetin	Leaf	[19]
17	Quercetin	Fruit, leaf, seed	[19,20,22]
18	Isorhamnetin	Fruit, seed	[20,22]
19	Hyperoside	Gall, leaf	[19]
20	Quercitrin	Leaf	[19]
21	Quercetin 3-glucoside (isoquercetin)	Leaf, seed	[19,21,22]
22	Quercetin 3-rutinoside (Rutin)	Fruit, gall, leaf, seed	[19,20,21,22]
23	Isorhamnetin-3-O-glucoside	Fruit, leaf, seed	[20,21,22]
24	Isorhamnetin 3-rutinoside	Fruit, leaf, seed	[20,21,22]
25	Isorhamnetin 3,7-diglucoside	Leaf, seed	[21,22]
26	Isorhamnetin 3-O-robinobioside	Seed	[22]
27	Isorhamnetin 3-O-galactoside	Seed	[22]
28	Epicatechin O-glucoside	Fruit	[20]
**COUMARINS**			
29	Bergapten	Stem bark	[23]
30	Marmesin	Stem bark	[23]
**ALKALOIDS**			
31	N-trans-Feruloyltyramine	Stem bark	[2]
32	N-cis-feruloyltyramine	Stem bark	[2]
33	Trigonelline	Fruit	[20]
**STEROIDS**			
34	Diosgenin	Fruit	[24]
35	Yamogenin	Fruit, root, stem bark	[25]
36	6-Methyldiosgenin	Fruit	[24]
37	Rotenone	Root	[26]
38	β-Sitosterol	Seed (oil)	[27]
39	Cholesterol	Seed (oil)	[27]
40	Campesterol	Seed (oil)	[27]
41	Stigmasterol	Seed (oil)	[27]
**PREGNANE GLYCOSIDES**			
42	Pregn-5-ene-3β,16β,20(R)-triol 3-O-(2,6-di-O-α-L-rhamnopyranosyl)-β-D-glucopyranoside	Fruit	[28]
43	Pregn-5-ene-3β,16β,20(R)-triol 3-O-β-D-glucopyranoside	Fruit	[28]
**SAPONINS**			
**SPIROSTANOL SAPONINS**			
44	Balanitin 1	Root, stem bark	[29]
45	Balanitin 2	Root, stem bark	[29]
46	Balanitin 3	Root, stem bark	[29]
47	Balanitin 4	Seed	[30]
48	Balanitin 5	Seed	[30]
49	Balanitin 6	Seed	[30,31]
50	Balanitin 7	Fruit, root, seed	[30,31,32]
51	Deltonin	Seed	[33]
52	(3β,20S,22R,25R)-spirost-5-en-3-yl β-D-xylopyranosyl-(1→3)-β-D-glucopyranosyl-(1→4)[α-L-rhamnopyranosyl-(1→2)]-β-D-glucopyranoside	Root	[34]
53	(3β,20S,22R,25S)-spirost-5-en-3-yl β-D-xylopyranosyl-(1→3)-β-D-glucopyranosyl-(1→4)[α-L-rhamnopyranosyl-(1→2)]-β-D-glucopyranoside	Root	[34]
**FUROSTANOL SAPONINS**			
54	Balanitesin	Fruit	[35]
55	Balanitoside	Fruit	[24,36]
56	22R and 22S epimers of 26-(O-β-D-glucopyranosyl)-3-β-[4-O-(β-D-glucopyranosyl)-2-O-(α-L-rhamnopyranosyl)-β-D-glucopyranosyloxy]-22,26-dihydroxyfurost-5-ene	Fruit	[37]
57	Xylopyranosyl derivative of 26-(O-β-D-glucopyranosyl)-3-β-[4-O-(β-D-glucopyranosyl)-2-O-(α-L-rhamnopyranosyl)-β-D-glucopyranosyloxy]-22,26-dihydroxyfurost-5-ene	Fruit	[37]
58	(3β,20S,22R,25R)-26-(β-D-glucopyranosyloxy)-22-methoxyfurost-5-en-3-yl β-D-xylopyranosyl-(1→3)-β-D-glucopyranosyl-(1→4) [α-L-rhamnopyranosyl-(1→2)]-β-D-glucopyranoside	Root	[34]
59	(3β,20S,22R,25S)-26-(β-D-glucopyranosyloxy)-22-methoxyfurost-5-en-3-yl β-D-xylopyranosyl-(1→3)-β-D-glucopyranosyl-(1→4) [α-L-rhamnopyranosyl-(1→2)]-β-D-glucopyranoside	Root	[34]
60	26-O-β-D-glucopyranosyl-(25R)-furost-5-ene-3β,22,26-triol 3-O-(2,4-di-O-α-L-rhamnopyranosyl)-β-D-glucopyranoside	Fruit	[38]
61	22-methyl ether of 26-O-β-D-glucopyranosyl-(25R)-furost-5-ene-3β,22,26-triol 3-O-(2,4-di-O-α-L-rhamnopyranosyl)-β-D-glucopyranoside	Fruit	[38]
62	26-O-β-D-glucopyranosyl-(25R)-furost-5-ene-3β,22,26-triol 3-O-[α-L-rhamnopyranosyl-(1→2)]-[β-D-xylopyranosyl (1→3)]-[α-L-rhamnopyranosyl-(1→4)]-β-D-glucopyranoside	Fruit	[38]
63	22-methyl ether of 26-O-β-D-glucopyranosyl-(25R)-furost-5-ene-3β,22,26-triol 3-O-[α-L-rhamnopyranosyl-(1→2)]-[β-D-xylopyranosyl (1→3)]-[α-L-rhamnopyranosyl-(1→4)]-β-D-glucopyranoside	Fruit	[38]
64	Balanin B2	Stem bark	[39]
65	26-(O-β-D-glucopyranosyl)-22-O-methylfurost- 5-ene-3β,26-diol-3-O-β-D-glucopyranosyl-(1→4)- [α-L-rhamnopyranosyl-(1→2)]-β-D-glucopyranoside	Fruit	[40]
66	25R and 25S epimers of 26-O-β-D-glucopyranosyl-furost-5-ene-3,22,26- triol 3-O-[α-L-rhamnopyranosyl-(1→3)- β-D-glucopyranosyl-(1→2)]- α-L-rhamnopyranosyl-(1→4)-β- D-glucopyranoside	Fruit	[41]
67	26-O-β-D-glucopyranosyl-(25R)-furost-5-ene-3,22,26-triol 3-O-[ β-D-glucopyranosyl-( 1→2)]- α-L-rhamnopyranosyl-(1→4)-β-D-glucopyranoside	Fruit	[41]
68	26-O-β-D-glucopyranosyl-(25R)-furost-5,20-diene-3,26-diol 3-O-[α-L-rhamnopyranosyl-(1→3)- β-D-glucopyranosyl-(1→2)]- α-L-rhamnopyranosyl-(1→4)-β-D-glucopyranoside	Fruit	[41]
69	25R and 25S epimers of 26-O-β-D-glucopyranosyl-furost-5,20-diene-3,26-diol 3-O-[ β-D-glucopyranosyl-(1→2)]- α-L-rhamnopyranosyl-(1→4)-β-D-glucopyranoside	Fruit	[41]
**OPEN-CHAIN STEROIDAL SAPONINS**			
70	(3β,12α,14β,16β)-12-hydroxycholest-5-ene-3,16-diyl bis (β-D-glucopyranoside)	Root	[34]
71	Balanin B1	Stem bark	[39]
72	β-Sitosterol glucoside	Stem bark	[42]
73	Stigmasterol-3-O-β-D-glucopyranoside	Stem bark	[40]

### 3.3. Coumarins

Coumarins are phenolic compounds displaying fused benzene and α-pyrone rings and are known for anti-inflammatory, anticoagulant, antimicrobial, anticancer, antioxidant and neuroprotective properties [43]. Bergapten (**29**) and marmesin (**30**) are coumarins extracted from stem bark (Table 5; Figure 3) [23]. 

### 3.4. Alkaloids

**Figure 4:**
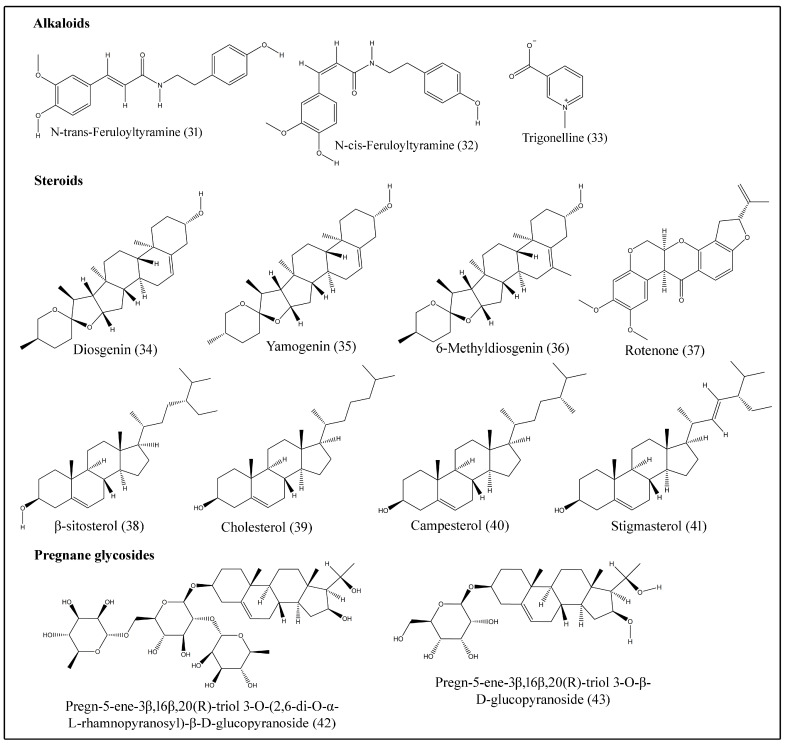
Structures of alkaloids, steroids and pregnane glycosides isolated from desert date.

Alkaloids are compounds that contain basic nitrogen atoms [44] and show varied biological activities. They are especially useful for cancer treatment. N-cis-feruloyltyramine (**32**), N-trans-feruloyltyramine (**31**) and trigonelline (**33**) are some of the alkaloids isolated from stem bark and fruit (Table 5; Figure 4) [2,20].

### 3.5. Phytosterols

Phytosterols are bioactive compounds found naturally in food with chemical structures similar to cholesterol. Various clinical studies consistently show that intake of phytosterols, such as beta-sitosterol, campesterol and stigmasterol, is associated with a significant reduction in levels of low-density lipoprotein in humans. *B. aegyptiaca* produces several steroids, such as campesterol (**40**), cholesterol (**39**), diosgenin (**34**), 6-methyldiosgenin (**36**), rotenone (**37**), β-sitosterol (**38**), stigmasterol (**41**) and yamogenin (**35**) (Table 5; Figure 4) [24,25,26,27].

Pregnane glycosides are naturally occurring sugar conjugates of C_21_ steroidal compounds, isolated from various plants and many show anticarcinogenic properties [45]. Pregn-5-ene-3β,16β,20(R)-triol 3-O-(2,6-di-O-α-L-rhamnopyranosyl)-β-D-glucopyranoside (**42**) and pregn-5-ene-3β,16β,20(R)-triol 3-O-β-D-glucopyranoside (**43**) were extracted from the fruits of desert date (Table 5; Figure 4) [28].

### 3.6. Saponins

**Figure 5:**
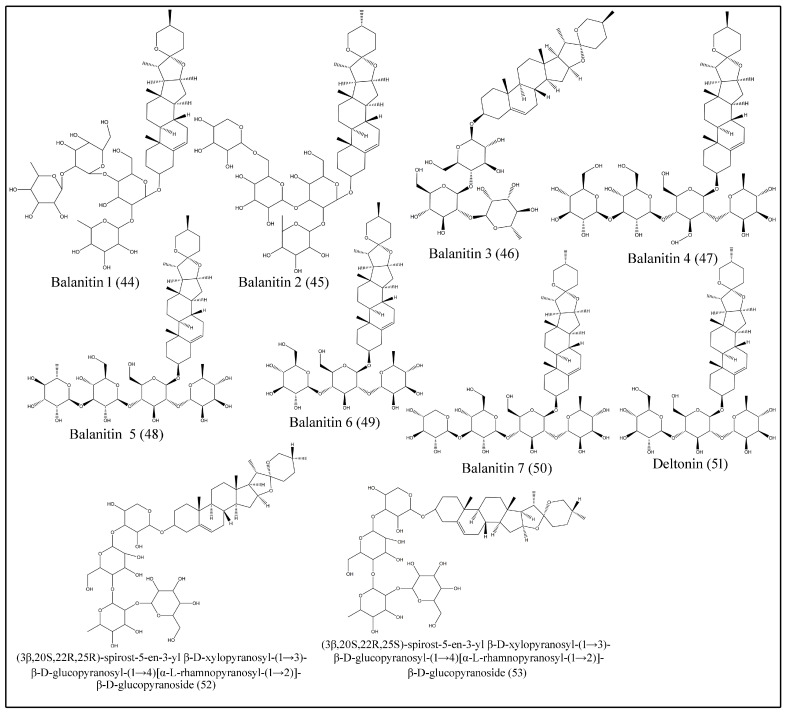
Structures of spirostanolsaponins isolated from desert date.

**Figure 6:**
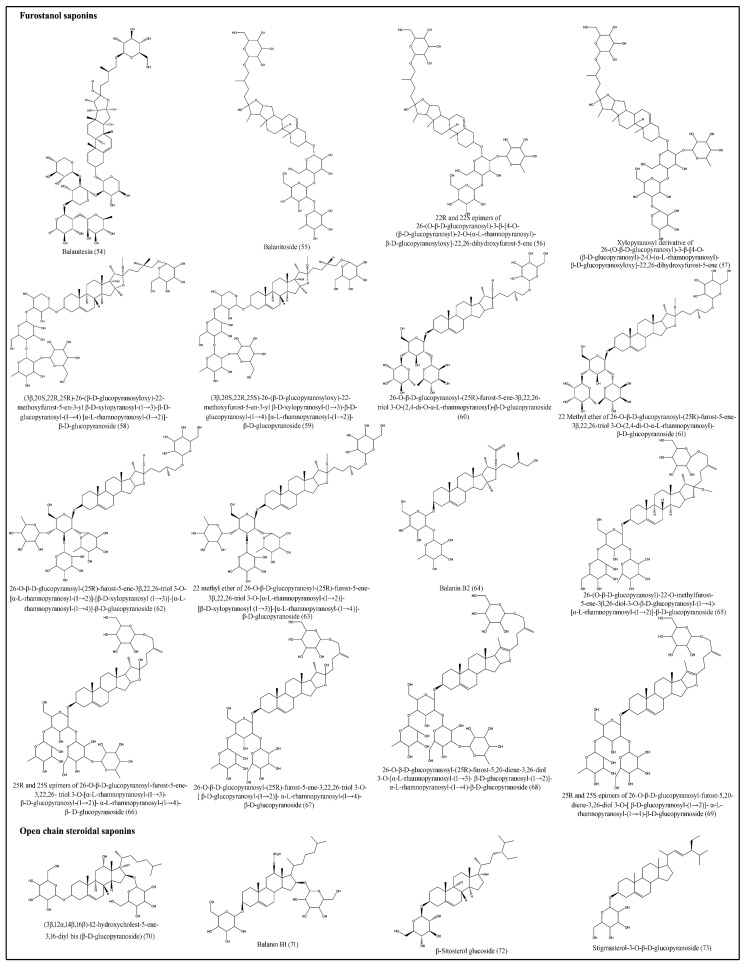
Structures of furostanoland open-chain steroidal saponins of desert date.

Saponins are bioorganic compounds that exhibit triterpenoid or steroidal skeletons that are glycosylated by varying numbers of sugar moieties attached at different positions. Steroidal saponins are further classified into spirostanol, furostanol and open-chain steroidal saponins [46]. Saponins exhibit a wide range of biological properties, including hemolytic factor sand anti-inflammatory, antimicrobial, insecticidal, anticancer and molluscicidal activities [47]. Various spirostanol, furostanol and open-chain steroidal saponins, which are isolated from fruits, seeds, roots and stem bark are presented in Table 5 and Figure 5 and Figure 6 [24,29,30,31,32,33,34,35,36,37,38,39,40,41,42].

## 4. Biological Activity

Extracts and compounds from extractions of *B. aegyptiaca* todate exhibit a wide range of biological activity (Table 6).

### 4.1. Antioxidant Properties

Various kinds of physical and physiological stresses lead to the overproduction of oxidants in the human body, which can cause oxidative damage of DNA, proteins and lipids. Furthermore, this damage is responsible for several disorders in the human body such as cardiovascular diseases, cancer and aging. It was reported that minor fruits and nuts possess abundant antioxidant phytochemicals, and the consumption of minor fruits and nuts is beneficial to the human body [48]. The antioxidant effects of methanol extracts of stem bark on 1,1-diphenyl-2-picrylhydrazyl (DPPH) and 2,2’-azino-bis(3-ethylbenzothiazoline-6-sulfonic acid (ABTS) scavenging is demonstrated and accounted for in the total soluble phenolic and flavonoid contents [49]. Furthermore, Hassan et al.’s [49] results show that methanol extracts display the highest phenolic content (35.17 mg gallic acid equivalents/g) and considerable flavonoid content (112.83 mg quercetin equivalents/g). Methanol extract showed the highest free radical scavenging activity at IC_50_ = 40 µg/mL and IC_50_ = 125.85 µg/mL in DPPH and ABTS assays, respectively. The antioxidant properties of aqueous fruit extracts were assessed in streptozotocin-induced diabetic rats [50]. Oral administration produced a significant (*p* < 0.01) increase in mean plasma total antioxidant levels and a significant (*p* < 0.01) decrease in malondialdehyde levels. The antioxidant properties of leaf and root extracts were also demonstrated [51]. Balanitin 1 and balanitin 2 (saponins) were isolated from bark extracts and demonstrated antioxidant properties in vitro, using a method based on the Briggs–Rauscher oscillating reaction [39]. Polyphenols such as quercetin and kaempferol are the major components responsible for antioxidant activities [52]. In addition, phytosterols including ß-sitoterol, stigmasterol and campesterol have been reported to exert antioxidant activity [53]. Polyphenols, phytosterols and saponins together might be responsible for the antioxidant activity of desert date.

### 4.2. Antimicrobial Properties

Plants synthesize several antimicrobial compounds, including phenolics such as simple phenols, phenolic acids, quinones, flavonoids, flavones, flavonols, tannins, coumarins, terpenoids, essential oils and alkaloids [54]. The mechanism of action of these compounds ranges from membrane disruption, substrate deprivation, intercalation into the cell wall/or DNA and enzyme inhibition. Desert date is rich in all these phytochemicals and demonstrates potent antimicrobial activity. The bark of *B. aegyptiaca* is widely used in African folk medicine for the treatment of wounds and skin diseases. The effects of aqueous ethanolic extracts of bark on bacteria isolated from wounds have been reported [55]. These extracts inhibited the growth of *Pseudomonas aeruginosa* and *Staphylococcus aureus* in vitro. The in vitro antifungal activity of saponin-rich extracts of fruit mesocarp was explored against phytopathogenic fungi [56]. These extracts were moderately active (34.7%) against *Alternaria solani* and highly active (89.01%) against *Pythium ultimum*, and activity was significantly higher compared to the fungicide, metalaxyl (15 μg/mL). The antifungal activity of ethanolic and methanolic extracts of root bark and fruit have been demonstrated against *Aspergillus niger*, *Candida albicans*, *Penicillium crustosum* and *Saccharomyces cerevisiae* [57].

### 4.3. Hepatoprotective Properties

A methanolic extract of leaves was evaluated for hepatoprotective activity against carbon tetrachloride (CCl_4_)-induced hepatic damage in rats [58]. Administration of the extract (200 and 400 mg/kg per os) markedly reduced the CCl_4_-induced elevation of serum marker enzymes, such as glutamate pyruvate transaminase, glutamate oxaloacetate transaminase, alkaline phosphatase and bilirubin. Similarly, fruit mesocarp and stem bark aqueous extracts ameliorated CCl_4_-induced hepatotoxicity in rats, as measured by liver enzyme activity, blood parameters and histopathology [59]. Ethanolic extracts of bark protected hepatocytes against paracetamol and CCl_4_-induced hepatotoxicity in rats, analogous to silymarin [60]. Bioactive compounds, primarily obtained from dietary sources, contain a wide range of free radical scavenging constituents, including polyphenols, alkaloids and phytosterols, which are responsible for hepatoprotective effects [61]. Desert date is rich in polyphenols, phytosterols and saponins. It has depicted very good antioxidant potential and thus increased the cellular antioxidant defense system, which may be responsible for the hepatoprotective effects of desert date.

### 4.4. Anticancer Properties

Cancer is a major health problem. Radiotherapy, chemotherapy and surgical removal are the current treatment methods. However, these methods have varied disadvantages such as drug resistance and toxic effects on nontargeted tissues. Therefore, researchers are searching for naturally available plant-based bioactive compounds for cancer therapy [62]. Among the plant-based bioactive compounds, saponins and phytosterols have significant importance in reducing the risk of cancer [63,64]. Various steroidal saponins isolated from various tissues of *B. aegyptiaca* are reported to display anticancer activities. For example, a mixture of balanitin-6 and balanitin-7 (28:72) isolated from kernels show growth inhibition in human cancer cell lines in vitro [31]. Balanitin-6/balanitin-7 exhibited higher antiproliferative activity than well-known natural cancer therapeutic agents, such as etoposide and oxaliplatin. Balanitin-6/balanitin-7 displayed its highest activity against A549 nonsmall cell lung cancer (IC_50_, 0.3 µM) and U373 glioblastoma (IC_50_, 0.5 µM) cell lines. Balanitoside extracted from the fruit also showed anticancer activity against Ehrlich ascites carcinoma (EAC)-bearing Swiss albino mice [36]. Mice injected intraperitoneally with balanitoside (10 mg/kg body weight) displayed decreases in liver and serum enzyme levels. Issa et al. [65] studied an aqueous extract of pulp on the development and growth of EAC and metastasis to the liver and spleen. Treatment with the extract (400 mg/kg) inhibited tumor growth and proliferation in ascetic fluid, inducing a significant decrease in tumor volume, total cell volume and viable cell count and prolonged mouse survival. The authors also recorded significant decreases in levels of lipid peroxidation and increased superoxide dismutase and catalase activity and P53 (a tumor suppressor protein) expression. The saponin, balanitin-7 isolated from seed kernels, showed antiproliferative activity [32]. These agents showed potent antiproliferative activity against MCF-7 human breast cancer cells and HT-29 human colon cancer cells, with IC_50_ values of 2.4 and 3.3 µM, respectively.

### 4.5. Anti-Inflammatory Properties

Inflammation is a pattern of response to injury, which involves the accumulation of cells, exudates in irritated tissue, which allows protection from further damage. A variety of in vitro and in vivo experiments has shown that certain flavonoids and saponins possess anti-inflammatory activity [66]. The mechanism by which flavonoids and saponins exert their anti-inflammatory effects involves the inhibition of cyclooxygenase and lipoxygenase activities [67]. Desert date exhibited potent anti-inflammatory activity; for example, Speroni et al. [39] studied the in vivo anti-inflammatory activity of methanol and butanol extracts and two saponins, viz. balanin-B1 and balanin-B2, isolated from *B. aegyptiaca* bark in rats with edema induced by carrageenin. Both extracts exhibited a significant reduction of rat paw edema. The inhibition produced by methanol extract, butanol extract, balanin-B1 and balanin-B2 were 32%, 68%, 62% and 59%, respectively. Likewise, the influence of seed oil on liver and kidney fractions in rat serum was evaluated [68]. Seed oil (100 mg/kg) in the rat diet decreased nitrogen oxide and lipid peroxidation. Further, mRNA and protein expression of tumor necrosis factor-α and interleukin-6 were downregulated, leading to a reduction of cyclooxygenase-2, reflecting anti-inflammatory activity.

### 4.6. Antidiabetic Activity

Diabetes is a chronic disease that occurs either when the pancreas does not produce enough insulin or when the body cannot effectively use the insulin it produces. Several medicinal plants have demonstrated hypoglycemic and hyperglycemic activities; these activities seem to be mediated through increased insulin secretion via stimulation of pancreatic cells, interfering with dietary glucose absorption or through insulin-sensitizing action [69]. Kamel et al. [38] demonstrated the antidiabetic effect of an aqueous extract of fruit in streptozotocin (STZ)-induced diabetic mice after oral administration. They also identified steroidal saponins, 26-O-ß-D-glucopyranosyl-(25R)-furost-5-ene-3ß,22,26-triol-3-O-[α-L-rhamnophyranosyl-(1→1)]-[ß-D-xylopyranosyl-(1→3)]-[α-L-rhamnopyronosyl-(1→4)]-ß-D-glucopyranoside and its 22-methyl ether in the extract and recognized two additional saponins, 26-O-ß-D-glycopyranosyl-(25R)-furost-5-ene-3ß,22,26,-triol-3-O-[2,4-di-O-α-L-rhamnopyranosyl)-ß-D-glucopyranoside and its methyl ether. A combination of saponins exhibited greater antidiabetic activity than individual saponins. Gad et al. [70] administered fruit extracts (1.5 g/kg body weight) to STZ-induced diabetic rats and studied the glycogen content of liver and kidney and on some key enzymes of liver involved in carbohydrate metabolism. STZ (50 mg/kg body weight) caused a five-fold increase in blood glucose level, an 80% reduction in serum insulin level, a 58% decrease in liver glycogen and a seven-fold increase in kidney glycogen content. A marked increment in the activity of glucose-6-phosphatase activity and decreased activity of glucose-6-phosphate dehydrogenase and phosphofructokinase were recorded. Treatment of rats with fruit extract reduced blood glucose levels by 24% and significantly decreased liver glucose-6-phosphatase activity. The authors also demonstrated that the extract inhibited α-amylase activity in vitro. The major component in the extract was diosgenin, based on high-performance thin-layer chromatography. Additionally, Al-Malki et al. [71] showed that ethyl acetate extract containing β-sitosterol modulated oxidative stress induced by streptozotocin.

**Table 6:** Biological activities of compounds isolated from various parts of desert date.

Compound	Part	Activity	Model/Method	Reference
Balanitin 1	Root and stem bark	Molluscicide	*Biomphalaria glabrata*	[29]
Balanitin 2	Root and stem bark	Molluscicide	*Biomphalaria glabrata*	[29]
Balanitin 3	Root and stem bark	Molluscicide	*Biomphalaria glabrata*	[29]
Balanitin 4	Seed	Anticancer	P-388 Lymphocytic leukemia cell line	[30]
Balanitin 5	Seed	Anticancer	P-388 Lymphocytic leukemia cell line	[30]
Balanitin 6	Seed	Anticancer	Different cancer cell lines including the P-388 Lymphocytic leukemia cell line and female mice injected with L1210 syngeneic murine leukemia cells	[30,31]
Balanitin 7	Fruit, root, seed	Anticancer	1.Different cancer cell lines and female mice injected with L1210 syngeneic murine leukemia cells 2. P-388 Lymphocytic leukemia cell line 3.Human breast cancer cells (MCF-7) and human colon cancer cells (HT-29)	[30,31,32]
		Nematocidal	*Caenorhabditis elegans*	[72]
Deltonin	Seed	Molluscicidal	*Biomphalaria glabrata*	[33]
Balanitoside	Fruit	Anticancer	Ehrlich ascites carcinoma bearing Swiss albino mice	[36]
		Antidiabetic	Streptozotocin-induced diabetes in Wistar rats	[73]
Balanin B2	Stem bark	Anti-inflammatory	Carrageenin-induced paw edema in male Sprague Dawley rats	[39]
26-(O-β-D-glucopyranosyl)-22-O-methylfurost- 5-ene-3β,26-diol-3-O-β-D-glucopyranosyl-(1→4)-[α-L-rhamnopyranosyl-(1→2)]-β-D-glucopyranoside	Fruit	Antidiabetic	α-Glucosidase and aldose reductase inhibitory activities (in vitro) and streptozotocin-induced diabetes in male albino Wistar rats (in vivo)	[40]
25R and 25S epimers of 26-O-β-D-glucopyranosyl-furost-5-ene-3,22,26- triol 3-O-[α-L-rhamnopyranosyl-( 1→3)- β-D-glucopyranosyl-(1→2)]- α-L-rhamnopyranosyl-(1→4)-β- D-glucopyranoside	Fruit	Aldose reductase inhibitor	Aldose reductase inhibition activity on rat liver homogenate	[41]
26-O-β-D-glucopyranosyl-(25R)-furost-5-ene-3,22,26-triol 3-O-[ β-D-glucopyranosyl-( 1→2)]- α-L-rhamnopyranosyl-(1→4)-β-D-glucopyranoside	Fruit	Aldose reductase inhibitor	Aldose reductase inhibition activity on rat liver homogenate	[41]
26-O-β-D-glucopyranosyl-(25R)-furost-5,20-diene-3,26-diol 3-O-[α-L-rhamnopyranosyl-(1→3)- β-D-glucopyranosyl-(1→2)]- α-L-rhamnopyranosyl-(1→4)-β-D-glucopyranoside	Fruit	Aldose reductase inhibitor	Aldose reductase inhibition activity on rat liver homogenate	[41]
25R and 25S epimers of 26-O-β-D-glucopyranosyl-furost-5,20-diene-3,26-diol 3-O-[ β-D-glucopyranosyl-(1→2)]- α-L-rhamnopyranosyl-(1→4)-β-D-glucopyranoside	Fruit	Aldose reductase inhibitor	Aldose reductase inhibition activity on rat liver homogenate	[41]
Balanin B1	Stem bark	Anti-inflammatory	Carrageenin-induced paw edema in male Sprague Dawley rats	[39]
		Antioxidant	ROS scavenging activity by Briggs-Rauscher oscillating reaction	[39]

Hassanin et al. [74] tested a crude ethanolic fruit extract and its butanolic and dichloromethane fractions on stress-activated protein kinase/c-Jun N-terminal kinase (SAPK-JNK) signaling in experimental diabetic rats. Six groups of male Wistar rats were used: normal control, diabetic, diabetic rats treated with crude, butanol or dichloromethane factions (50 mg/kg body weight), and diabetic rats were treated with gliclazide as a reference drug. Treatments continued for one month. Extract treatments produced a reduction in plasma glucose, hemoglobin A1c, lactic acid, lipid profile and malondialdehyde levels, which induced an increase in insulin and reduced glutathione (GSH) levels and catalase and superoxide dismutase activities. Moreover, the authors observed the downregulation of apoptosis signal-regulating kinase 1, c-Jun N-terminal kinase 1 and protein 53 and the upregulation of insulin receptor substrate 1 in rat pancreas. Glucose transporter 4 was upregulated in rat muscle. Liquid chromatography and high-resolution mass spectrometry (LC-HRMS) analysis identified balanitin-2, hexadecenoic acid, methyl protodioscin and 26-(O-β-D-glucopyranosyl)-3-β-[4-O-(β-D-glucopyranosyl)-2-O-(α-L-rhamnopyranosyl)-β-D-glucopyranosyloxy]-22,26-dihydroxyfurost-5-ene in crude extract and balanitin-1 and trigonelloside C in butanol and dichloromethane fractions of crude extract. Ezzat et al. [40] isolated several compounds from pericarp, including stigmasterol-3-O-β-D-glucopyranoside (**a**), a pregnane glucoside: pregn-5-ene-3β,16β,20(R)-trio1-3-O-β-D-glucopyranoside (**b**); a furostanol saponin: 26-(O-β-D-glucopyranosyl)-22-O-methylfurost-5-ene-3β,26-diol-3-O-β-D-glucopyranosyl-(1→4)-[α-L-rhamnopyranosyl-(1→2)]-β-D-glucopyranoside (**c**). The latter component possessed significant α-glucodidase (AG) and aldose reductase inhibitory activities in streptozotocin-induced diabetic Wistar rats. Compound (**c**) also caused a significant increment in insulin and C-peptide levels.

### 4.7. Molluscicidal Activity

Regarding the effects of fruit extracts on juvenile and adult *Bulinus globosus* and *B. truncatus,* two Planorbid (ramshorn) freshwater snails have been reported [75]. LC_95_ values were 16.9 and 19.7 μg/mL and 14.2 and 12.0μg/mL for juvenile and adults of *B. globosus* and *B. truncatus*, respectively. Seed, endocarp, mesocarp and whole fruit extracts were assessed against adult *Biomphalaria pfeifferi,* another Planorbid snail, and *Lymnaea natalensis,* a Lymnaeid pond snail [76]. LC_90_ values were 77.70, 120.04, 89.50 and 97.55 mg/L against *Biomphalariapfeifferi* for seed, endocarp, mesocarp and whole fruit extracts, respectively, and 102.30, 138.21, 115.42 and 127.69 mg/L against *Lymnaeanatalensis.* Furthermore, the molluscicidal activity of seed oil on *Monacha cartusiana*, a Hygromiid land snail, has been demonstrated [77]. Bioactive compounds were identified as saponins, such as diosgenin, yamogenin and 3,5-spirostadiene.

### 4.8. Other Activities

Several studies demonstrate the antinematode and antiplasmodial activities of *B. aegyptiaca* extracts. Shalaby et al. [78] showed the effects of methanolic fruit extracts on enteral and parenteral stages of *Trichinella spiralis* (pork worm). The authors also evaluated the effectiveness of methanolic extract against preadult migrating larvae and encysted larvae of *Trichinella spiralis* in rats and compared them with the commonly used anthelmintic chemical, albendazole. Methanolic extract (1000 mg/kg body weight) for five successive days throughout the parasite lifecycle led to a marked reduction in migrating and encysted larvae by 81.7% and 61.7%, respectively. In another study, the efficacy of a methanolic extract on *Toxocara vitulorum* (roundworm), a major parasite in cattle and buffalo, was assessed [79]. They incubated parasites in a ringer solution containing 10, 30, 60, 120 and 240 µg/mL of ethanolic extract for 24 h. The most prominent activity at 240 µg/mL caused the disorganization of body cuticle musculature. Kusch et al. [17] evaluated a crude extract of seeds for antiplasmodial activity. An IC_50_ value for chloroquine-susceptible *Plasmodium falciparum* NF54 was 68.26 µg/µL. The compound responsible for this activity was 6-phenyl-2(H)-1,2,4-triazin-5-one oxime. The authors also showed that two phenolic compounds, 2,6-di-*tert*-butyl-phenol and 2,4-di-*tert*-butylphenol, displayed antiplasmodial activity at IC_50_ values of 50.29 and 47.82 µM, respectively.

## 5. Conclusions

*B. aegyptiaca* or desert date is an underutilized tree species. The nutritional status of the fruits, leaves and seeds indicate that this species could be exploited as a food source. Seed oil might also be a good source of biodiesel. Leaves and young shoots are nutritionally rich and could be exploited as cattle feed. Furthermore, fruits, leaves, roots and the bark of stem and roots are substantial sources of bioactive phytochemicals that display a host of possibly useful biological properties. *B. aegyptiaca* might prove to be a valuable source of bioactive agents for use in human and veterinary medicine.
